# Indents

**Published:** 1923-01

**Authors:** 


### INDENTS

**☞Brief Articles of Technical or Scientific Value will receive notice and comment. Contributions invited.**

---

Mercurophen and Mercurochrome as Germicidal and Antiseptic Preparations. In the department of medicine there has recently been brought forward the two valuable preparations named above. Of the two, Mercurophen, according to the reports of the medical profession, is a much more stable product and in fact the dilute solution in pure distilled water may be kept indefinitely in tightly stoppered bottles without loss of germicidal power. Exposure to light has no effect on the solution; but care must be taken to prevent evaporation.

These preparations, from what I have learned, are superior to Dakin's solution on account of being more stable. As far as penetrating properties are concerned, they are superior to Dakin's.

Mercurophen (sodium-oxymercury-ortho-nitro-phenolate) is a development of the Dermatological Research Institute of Philadelphia and is the result of the investigation of Drs. Schamberg, Raezess and Kolmer of Philadelphia.

Both preparations are powerful germicides and antiseptics belonging to the mercurial group, but with advantages greatly surpassing bichlorid of mercury, although Mercurophen contains only 50 per cent. mercury and Mercurochrome from 23 to 24 per cent.

It appears that Mercurophen has a selective affinity for the staphylococci, streptococci and spore-forming bacilli. The phenol coefficient of Mercurophen varies from 900 to 1300 and as to its power of precipitating albumen, it is about one-fourth to one-fifth that of mercuric chlorid. From this it can be seen that its irritating properties are very little or practically *nil*. In dilutions of 1:200 it has no effect on serum. On account of its low protein precipitating power and its high germicidal action, Mercurophen is of great value for sterilization of infections about the mouth and especially valuable in root-canal therapy. It is also of value in sterilization of instruments; that is, if one wishes to immerse them in a tray containing a germicidal solution. It is odorless and does not tarnish steel, brass, nickel or silverplated instruments. For instruments or surgeon's gloves a 1:5000 solution for one minute is sufficient. For disinfection of hands or field of operation a 1:1000 for one minute is sufficient.

Mercurochrome (disodium salt of dibrom-oxymercury-fluorescin) is manufactured by Hynson, Westcott & Dunning, chemists of Baltimore, Md. This latter preparation exhibits about the same phenomena as Mercurophen. Both are practically non-irritating, a 1 per cent. solution having been used by ophthalmologists in the treatment of diseased conditions of the eye and in general surgery. Reports indicate that Mercurochrome is very valuable in dressing open wounds and sinuses. It is reported to be most efficient in disinfecting the throats of diphtheria carriers. One prominent surgeon expresses himself as follows: "It is proved to be a non-irritating germicide which is effective even in the presence of serum, which does not require many minutes to manifest its germicidal activity and which will penetrate into the mucous membrane to an unusual degree."

Since in the treatment of root canals, a preparation is required that is non-irritating, non-coagulating and one that will penetrate deeply in the presence of serum, I believe that either product named deserves some consideration in the field of dental therapeutics. As far as root-canal treatment is concerned, I would advise that they be used on posterior teeth only, for these preparations will produce a stain, though not a permanent one. But for the treatment of wounds about the oral cavity and infected sockets I believe the stain has an advantage, for it shows to what depth the preparation has penetrated.—George J. Bleecher, D. D. S., in *Dental Cosmos.*

---

Analgesic Value of Aspirin when Applied Locally.

It has been found by many nose and throat surgeons in this region that the insufflation of powdered aspirin for the subsequent pain following various throat operations has been productive of greater relief to patients than application of procain or cocain, and without a possible poisoning of the regional tissues and retardation of healing.

This knowledge has led me to use an aqueous paste of aspirin for the packing of painful sockets following extractions, and as a sedative and analgesic in partially erupted third molar teeth; packing the paste gently and carefully into the pocketed gum and well over the region affected.

Aspirin is antiseptic and germicidal, thus filling a dual role and all with a markedly beneficial effect.—S. Loebenstein, D. D. S., in *Dental Cosmos.*

---

To Remove Cloth from Rubber in Packing. Apply cold water to the surface of the cloth and stroke the surface lightly with the ends of the fingers, which will cause the rubber to recede from the meshes of the cloth while you pull the cloth away.—W. E. McQueen, D. D. S., in *Dental Cosmos.*

---

Mercurochrome in Stomatitis. Ten per cent. alcoholic sol. mercurochrome offers great possibilities in ulcerative conditions of the mouth; as a post-operative application following surgical interference, stimulating and hastening cell proliferation, and likely as a remedy in Vincent's infection, apparently inhibiting the growth of the organisms involved—Vincent's spirillum and Bacillus fusiformis. It seems to impart an immediate betterment to injured ulcerous gum tissues and mucous membrane.—S. Loebenstein, D. D. S., in *Dental Cosmos.*

